# Podocyte cell-specific *Npr1* is required for blood pressure and renal homeostasis in male and female mice: role of sex-specific differences

**DOI:** 10.1152/physiolgenomics.00137.2023

**Published:** 2024-08-05

**Authors:** Chandramohan Ramasamy, Kandasamy Neelamegam, Samivel Ramachandran, Huijing Xia, Daniel R. Kapusta, Farhad R. Danesh, Kailash N. Pandey

**Affiliations:** ^1^Department of Physiology, School of Medicine, Tulane University Health Sciences Center, New Orleans, Louisiana, United States; ^2^Department of Pharmacology, Louisiana State University Health Sciences Center, New Orleans, Louisiana, United States; ^3^Section of Nephrology, Division of Internal Medicine, The University of Texas MD Anderson Cancer Center, Houston, Texas, United States

**Keywords:** conditional knockout, GC-A/NPRA, natriuretic peptides, podocyte, salt diet and blood pressure

## Abstract

Atrial and brain natriuretic peptides (ANP and BNP) bind to guanylyl cyclase A/natriuretic peptide receptor A (GC-A/NPRA), stimulating natriuresis and diuresis and reducing blood pressure (BP), but the role of ANP/NPRA signaling in podocytes (highly specialized epithelial cells covering the outer surfaces of renal glomerular capillaries) remains unclear. This study aimed to determine the effect of conditional deletion of podocyte-specific *Npr1* (encoding NPRA) gene knockout (KO) in male and female mice. Tamoxifen-treated wild-type control (PD *Npr1* f/f; WT), heterozygous (PD-Cre-*Npr1* f/+; HT), and KO (PD-Cre-*Npr1* f/−) mice were fed a normal-, low-, or high-salt diet for 4 wk. Podocytes isolated from HT and KO male and female mice showed complete absence of *Npr1* mRNA and NPRA protein compared with WT mice. BP, plasma creatinine, plasma sodium, urinary protein, and albumin/creatinine ratio were significantly increased, whereas plasma total protein, albumin, creatinine clearance, and urinary sodium levels were significantly reduced in the HT and KO male and female mice compared with WT mice. These changes were significantly greater in males than in females. On a normal-salt diet, glomerular filtration rate was significantly decreased in PD *Npr1* HT and KO male and female mice compared with WT mice. Immunofluorescence of podocin and synaptopodin was also significantly reduced in HT and KO mice compared with WT mice. These observations suggest that in podocytes, ANP/NPRA signaling may be crucial in the maintenance and regulation of glomerular filtration and BP and serve as a biomarker of renal function in a sex-dependent manner.

**NEW & NOTEWORTHY** Our results demonstrate that the podocyte-specific deletion of *Npr1* showed increased blood pressure (BP) and altered biomarkers of renal functions, with greater magnitudes in animals fed a high-salt diet in a sex-dependent manner. The results suggest a direct and sex-dependent effect of *Npr1* ablation in podocytes on the regulation of BP and renal function and reveal that podocytes may be considered an important target for the ANP-BNP/NPRA/cGMP signaling cascade.

## Graphical abstract

**Figure:**
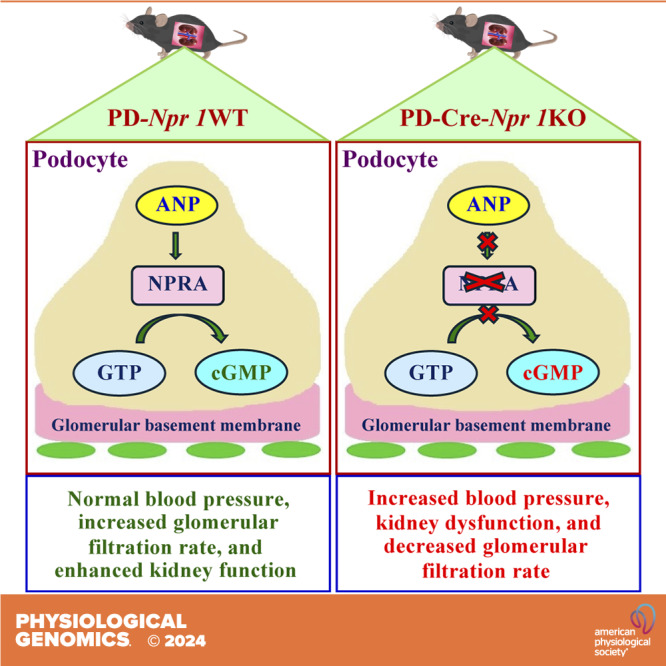

## INTRODUCTION

The cardiac hormones atrial natriuretic peptide and brain natriuretic peptide (ANP and BNP, respectively) bind to the transmembrane guanylyl cyclase A/natriuretic peptide receptor A (GC-A/NPRA) and generate a second messenger, cGMP, which reduces blood pressure (BP) and blood volume (1–4). Natriuretic peptides and their receptors play protective roles against cardiac, renal, and vascular dysfunction, and the ANP-BNP/NPRA/cGMP cascade regulates multiple physiological outputs, including natriuresis, diuresis, vasodilation, and the inhibition of the renin-angiotensin-aldosterone system (RAAS) (5–8). GC-A/NPRA is a transmembrane protein encoded by the *Npr1* gene and expressed in multiple organs; in the kidneys, it is expressed in renal juxtaglomerular cells, renal arterioles, podocytes, mesangial cells, proximal and distal tubules, ascending loops of Henle, and collecting duct (9, 10).

Infusion of ANP/BNP decreases serum creatinine levels and increases glomerular filtration rate (GFR), urine volume, and creatinine clearance (CrCl) in patients with normal renal function (11, 12). Infusion of either ANP or BNP in patients with preoperative renal dysfunction attenuates the increase in serum creatinine levels and maintains CrCl and GFR, preventing kidney dysfunction (13). ANP also enhances CrCl and GFR in patients with acute kidney injury after cardiac surgery (14). The ANP analog nesiritide attenuates major adverse cardiovascular events (MACE), including ejection fraction and survival, during cardiac surgery in patients with left ventricular dysfunction (15). The administration of nesiritide in patients with MACE also improved renal function, with better preservation of GFR and greater urine output. ANP directly increases the glomerular capillary ultrafiltration constant by inducing relaxation of the contractile intraglomerular mesangial cells between the capillary endothelium and podocytes, which increases the capillary surface area for filtration (16). The kidney glomerulus is a specialized structure that filters blood and retains essential plasma proteins. The glomerular filtration barrier is also a specialized capillary wall comprising fenestrated endothelial cells, podocytes, and an intervening basement membrane. Podocytes, specialized epithelial cells attached to the glomerular basement membrane (GBM), are an essential part of the glomerular filter barrier preventing the loss of serum proteins into the urine (17). In renal diseases, podocytes are injured or even lost, blocking the filtration of blood and leading to proteinuria (18, 19). Podocytes play a crucial role in barrier function and in the pathogenesis of glomerular diseases, forming a branched interlocked network with foot processes by the slit diaphragm (SD) (20).

Nephrin, podocin, and synaptopodin are the critical components of the SD between foot processes, maintaining the integrity and normal function of the filtration barrier. Nephrin, a key transmembrane protein with both extracellular and intracellular domains, forms the scaffolding of the podocyte SD. Podocin is also a transmembrane portion, which is expressed only in the podocytes and serves as a scaffolding protein in the SD complex (21). Podocin-nephrin interaction is important for nephrin recruitment to the lipid raft and signaling network (22–24). Synaptopodin, an actin-associated protein expressed in podocyte foot processes, interacts with α-actin and plays a role in the elongation of actinin filaments and bundling (25, 26). Genetic studies have established the roles of SD-associated proteins, including nephrin, podocin, and synaptopodin, in various proteinuric disorders (27). NPRA is highly expressed in glomerular podocytes compared with low expression of natriuretic peptide receptor-B (NPRB) (28). ANP/NPRA signaling in kidneys counter regulates angiotensin II (ANG II) signaling pathways (29–33), and a number of studies have indicated that ANP-BNP/NPRA/cGMP signaling exerts podocyte protective effects in renal disorders and disease, including aldosterone-induced podocyte injury, immune-mediated renal injury, and diabetic nephropathy (9, 34–36). We have previously demonstrated that global *Npr1*-disrupted heterozygous 1-copy mice fed a high-salt diet showed elevated systolic BP (SBP) and aggravated levels of cardiac ANG II, aldosterone, and proinflammatory cytokines, whereas *Npr1* gene-duplicated mice on the same diet did not render such elevated effects (37, 38). Our previous studies also found essential roles for GC-A/NPRA signaling in BP regulation, acute volume handling, and protecting the heart from salt loading through the kidneys (39–42), but the role of GC-A/NPRA in podocytes is unclear.

A growing body of evidence suggests that sex hormone levels and receptor density can significantly alter renal hemodynamics and BP homeostasis in a sex-dependent manner (43, 44). In humans (45) and genetic models of experimental hypertension such as spontaneously hypertensive rats (SHRs) (46), males develop earlier and more severe high BP than females. Previous studies have suggested that the progression of renal disease is more pronounced in men than in women, and men seem to have more severe end-stage kidney disease (ESKD) than women (47–50), but the mechanisms driving sex-specific differences in renal pathology and dysfunction are not well known. Sex hormone-specific intracellular milieu might contribute to divergent responses between sexes, including sex differences on the effects of cell-specific inactivation of *Npr1* in the regulation of high BP and renal dysfunction under physiological or pathological conditions in vivo. Genes and genetic mechanisms significantly impact BP and kidney disorders (51). To explore the role of genetic disruption of *Npr1* in podocytes, we used Cre/LoxP methodology to generate mutant mice with podocyte cell-specific disruption of *Npr1*. We performed BP and renal functional studies in mice with podocyte-specific *Npr1* disruption and challenged both sexes with low-salt (LS) and high-salt (HS) diets to determine whether a podocyte-specific inactivation of *Npr1* alters hypertension and renal dysfunction in a sex-dependent manner.

## MATERIALS AND METHODS

### Materials

Tamoxifen was purchased from Gojira Fine Chemicals (Bedford Heights, OH). Fluorescein isothiocyanate (FITC)-sinistrin was obtained from MediBeacon (St. Louis, MO). Salt diets were purchased from Teklad (Indianapolis, IN). The primary antibody of podocin (bs-6597R-FITC) was obtained from Bioss Antibodies (Woburn, MA), and synaptopodin antibody (sc-515842- FITC) was obtained from Santa Cruz Biotechnology (San Diego, CA). 4′,6-Diamidino-2-phenylindole (DAPI) was purchased from Vector Laboratories (Burlingame, CA). NPRA antibody was raised in our laboratory using a custom service from Genway Biotech (San Diego, CA). To measure albumin, an enzyme-linked immunosorbent assay (ELISA) kit was obtained from Bethyl Laboratories (Montgomery, TX). The quantichrome BCG albumin assay kit, quantichrome total protein assay kit, and enzymatic creatinine assay kit (EnzyChrom) were purchased from BioAssay Systems (Hayward, CA). All other chemicals were molecular biology reagent grade and were obtained from certified vendors.

### Construction of Conditional Targeting Vector for the Generation of Floxed *Npr1* Mice

**Figure 1.**
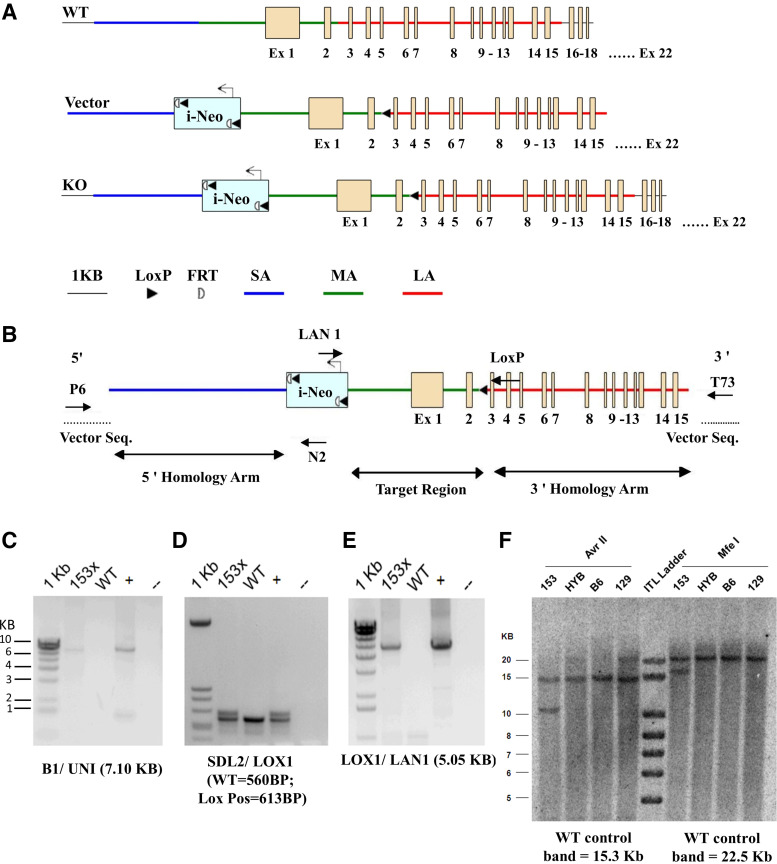
Design of the conditional targeting vector construct and the recombinant homologous allele and confirmation of distal LoxP retention by PCR using the LOX1 and LAN1 primers. *A* and *B*: design of the targeting vector with a restriction map of the partial mouse *Npr1* locus. *C*: *clone 153* was identified as a positive clone of interest, selected for expansion, and reconfirmed for SA integration. x denotes the expanded clone. DNA from the individual clone (before expansion) was used as a positive control and denoted with +. A negative control was used without DNA and denoted by a dotted line. Wild-type DNA was also used as a negative control. *D*: PCR was performed on *clone 153* to detect the presence of the distal LoxP site using the SDL2 and LOX1 primers and amplified a WT product 560 bp in size. The presence of a second PCR product 613 bp greater than the WT product indicated a positive LoxP site. *E*: distal LoxP retention was confirmed by PCR using the LOX1 and LAN1 primers, which produced a product 5.05 kb in size. *F*: for Southern blot analysis, DNA was digested with AvrII and MfeI. The DNA from C57Bl/6 (B6), J129/SvEv (J129), and BA1 (C57Bl/6 x J129/SvEv) (hybrid) mouse strains was used as WT controls with the expected sizes of 15.3 and 22.5 kb and targeted allele with expected sizes of 10.4 and 16.9 kb, respectively, to AvrII and MfeI. KO, knockout; WT, wild-type.

A 17.47-kb genomic region was selected to construct the targeting vector and subcloned from a positively identified C57Bl/6 BAC clone. The genomic region was designed with the 5′ homology arm, which extends 6.00 kb 5′ to the LoxP-FRT flanked Neo cassette. The 3′ homology arm ends 3′ to the single LoxP site, which is 7.04 kb long. The LoxP-FRT-PGK-gb2-Neo-FRT cassette was inserted into 2.15 kb 5′ of exon 1. The single LoxP site, containing engineered ApaLI and NheI sites for Southern blot analysis, was inserted 3′ downstream of exon 2. The target region consisted of 4.43 kb and included both exons 1 and 2 (Fig. 1*A*). The targeting vector was confirmed by restriction analysis after each modification step. P6 and T73 primers annealed to the backbone vector sequence and read into the 5′ and 3′ ends of the BAC subclone. LAN1 and N2 primers annealed to the 5′ and 3′ ends of the LoxP-FRT-PGK-gb2-Neo-FRT cassette, respectively. The single LoxP site was confirmed by sequencing primer Lox1. The total size of the targeting construct (including vector backbone and Neo cassette) was 23.61 kb (Fig. 1*B*). The screening primer B1, designed upstream of the short homology arm (SA) outside the 5′ region, was used to generate the targeting construct. PCR using B1 with the UNI primer (located within the Neo cassette) was used to amplify 7.10 kb fragments (Fig. 1*C*). The targeting construct was linearized using NotI prior to electroporation into embryonic stem (ES) cells. Ten micrograms of the linearized targeting vector were then transfected by electroporation of BA1 (C57Bl/6 x J129/SvEv) (hybrid) ES cells. After selection with G418 antibiotic, the surviving clones were expanded for PCR analysis to identify recombinant ES clones. *Clone 153* was identified as positive, selected for expansion, and reconfirmed for SA integration. PCR was performed on *clone 153* to detect the presence of the distal LoxP site using the SDL2 and LOX1 primers, and this reaction amplified a WT product 560 bp in size (Fig. 1*D*). A second PCR identified a 613-bp product greater than the WT product that indicated a positive LoxP PCR. Confirmation of distal LoxP retention was performed by PCR using the LOX1 and LAN1 primers, resulting in a product 5.05 kb in size (Fig. 1*E*). Sequencing was performed on purified PCR DNA to confirm the presence of the distal LoxP cassette using the SDL2 primer. The cassette containing distal LoxP and an engineered restriction site was as follows: 5′-CTAGCATAACTTCGTATAGCATACATTATACGAAGTTATGTGCACGTACGTGC-3′. The identified positive clone was subjected to Southern blot analysis to confirm the integration of 5′ and 3′ homology arms (Fig. 1*F*).

### Implantation of ES Cells Into Blastocysts and Production of Podocyte-Specific *Npr1* Knockout Mice

ES cells from recombinant *clone 153* were injected into mouse CD1 blastocysts and transferred into pseudo-pregnant CD1 females [custom service from the Ingenious Targeting Laboratory (iTL), Ronkonkoma, NY]. Four highly chimeric males were generated from the ES cell injection. The chimeras were mated with C57Bl/6 FLP deleter mice to generate somatic neo-deleted heterozygous mice, which were then mated with C57Bl/6 WT mice to produce germline neo-deleted heterozygous mice. These mice were mated to each other to generate homozygous flox/flox (f/f) mice. Podocyte-specific *Npr1* KO mice were generated by breeding *Npr1* f/f mice with tamoxifen-inducible heterozygous podocin (Nphs2) Cre mice (*Npr1* f/f x Nphs2-Cre). Nphs-Cre mice were generated as previously described (52). All mice used in the present study were on a hybrid mixed background (∼75% C57Bl/6 and ∼25% J129).

### Generation and Genotyping of Mice

Mice with a floxed *Npr1* allele were bred to Podo Cre mice, thereby deleting *Npr1* selectively in podocytes. The mice were maintained at the Tulane University School of Medicine Vivarium. Animals were handled according to the protocols of the approved study by the Institutional Animal Care and Use Committee. Mouse colonies were housed under 12:12-h light-dark cycles at 25°C and fed regular chow (Purina Laboratories, Framingham, MA), and tap water was available ad libitum. Mice were genotyped for *Npr1*^flox^ allele using forward primer 5′-TGAGAT-TTG-GAG-CCC-AGG-TGT-AGG-3′ and reverse primer 5′-GGCAGC-CTA-AGG-AAG-GAA-TCA-TTG-3′ (614-bp product for the *Npr1* floxed allele and 561-bp product for the WT allele) and for Nphs2-Cre+ using forward primer 5′-TCA-ACA-TGC-TGC-ACA-GGA-GAT-3′ and reverse primer 5′-ACCATA-GAT-CAG-GCG-GTG-GGT-3′ (800-bp product for Nphs-Cre and 500-bp product for WT mice). The animals were genotyped using DNA isolated from tail biopsies, and PCR was performed using our previously published standard methods (40, 53). All animals were littermate progeny of the same genetic background and were designated as podocyte-specific *Npr1* gene-disrupted heterozygous/haplotype (PD-Cre-*Npr1* f/+; HT), *Npr1* KO (PD-Cre-*Npr1* f/−; KO), or *Npr1* WT control (*Npr1* f/f; WT) mice. This study used adult (12–16 wk) male and female mice.

### Measurement of BP by Radiotelemetry and Computerized Tail-Cuff Methods

Mean arterial pressure (MAP) was recorded for 12 wk in chronically catheterized WT and KO mice placed on a normal-salt (NS) diet using radiotelemetry (Data Sciences International, New Brighton, MN) as previously described (54, 55). The transmitter was turned on magnetically for 24 h and soaked in sterile saline solution for at least 10 min prior to surgery. The production of radiotransmission was confirmed with an AM radio set at a low frequency. The mice were anesthetized with isoflurane (3–5% induction and 0.5–1% maintenance). A 50:50 solution of bupivacaine and lidocaine was administered subcutaneously for analgesia at the surgical site prior to incision. The left carotid artery was exposed, isolated, and temporarily occluded. The artery was cannulated with the catheter portion of the transmitter (model TA11PH-10, Data Sciences International), and the main body of the device was secured inside a skin pocket fashioned on the right flank. The skin was sutured, and the mice were closely observed and kept warm until recovery. The analgesic buprenorphine (Buprenex, 0.05–0.1 mg/kg body wt) was given subcutaneously to alleviate postoperative pain and distress, and mice were monitored for any discomfort, distress, or pain. The animals were moved to a cage placed over the receiver board. A computer program (Dataquest, St. Paul, MN) provided continuous monitoring and recording of all data at predetermined intervals. Mice were allowed to recover for 7 days with a full return to normal activity before measurements were taken. BP waveforms were sampled at a rate of 1,000 Hz for 10 s every 10 min. MAP was averaged weekly during light (6:00 AM–6:00 PM) and dark (6:00 PM–6:00 AM) periods to record BP in PD-*Npr1* HT, KO, and WT male and female mice throughout the 12-wk study. Experiments were initiated at the same time each day to prevent any diurnal variation in BP measurements. SBP was measured by noninvasive computerized tail-cuff method (Visitech 2000) as previously reported (56). BP was measured and calculated as the average of six sessions per day, and mice were trained for 5 consecutive days. Littermates of the same genotype and sex but without tamoxifen treatment were used as controls.

### Experimental Animal Groups and Metabolic Studies

Adult PD-*Npr1* HT, KO, and age-matched littermate WT male and female mice were intraperitoneally injected with tamoxifen (20 mg/day in corn oil) on 5 consecutive days to inactivate *Npr1* followed by a 10-day off period, after which all experiments commenced. Mice were randomly divided into six cohorts: WT mice treated with vehicle (corn oil), WT mice treated with tamoxifen, *Npr1* HT mice treated with vehicle, *Npr1* HT mice treated with tamoxifen, *Npr1* KO mice treated with vehicle, and *Npr1* KO mice treated with tamoxifen. Mice were fed for 28 days with different salt diets (Teklad): NS (0.3% NaCl), LS (0.05% NaCl), and HS (4% NaCl). SBP was measured in conscious mice using a noninvasive computerized tail-cuff method (Visitech 2000) at 0, 1, 2, 3, and 4 wk after rigorous tail-cuff training for 5 days as previously described (56, 57). Most other measurements were taken at *day 28*. In the preliminary studies, we determined baseline differences in genotypes before tamoxifen treatment, but none was found. On the 28th day, mice were placed individually in MMC100 metabolic cages (Hatteras Instruments, Grantsboro, NC) for 24 h to record food intake and to collect excreted urine. There was an adaptation of 1 day for animals to the metabolic chambers. Total urine output and albumin, creatinine, and sodium concentrations were measured from the cumulative urine excreted during the 24-h protocol. Urine volumes were recorded and kept at −20°C until used. The body weight of each mouse was measured. At the end of the experiment, animals were euthanized using a high concentration of CO_2_ gas, blood was collected, and the heart and kidneys were removed and weighed from the PD-*Npr1* KO, and WT mice and used for biochemical, molecular, and immunofluorescence analyses. To avoid the possible influence of covariates in the current experiments, mice were randomly allocated to treatment and control groups. Power analysis was used to determine the appropriate animal number for the experiments.

### Measurement of Albumin, Creatinine, Sodium, and Total Protein in Urine and Plasma

Urinary albumin excretion was measured in 24-h urine samples collected from mice in a metabolic cage using an ELISA kit (Bethyl Laboratories). Plasma albumin levels were measured using a quantichrom BCG albumin assay kit, and total protein in urine and plasma was assayed using a quantichrome total protein assay kit from BioAssay Systems (Hayward, CA) following the manufacturer’s instructions. Plasma and urine creatinine concentrations were determined using an enzymatic creatinine assay kit (EnzyChrom, BioAssay Systems) following the manufacturer’s protocol. The rate of CrCl was calculated from the creatinine concentration in the 24-h collected urine and plasma samples. The urine volume was recorded as mL/24 h/g body wt. The albumin/creatinine ratio was calculated from their respective concentrations measured in urine samples. Plasma and urinary sodium were measured using Flame Photometer IL973 (Instrumentation Laboratory, Lexington, MA).

### Measurement of Transcutaneous Glomerular Filtration Rate by the MediBeacon Device in Conscious Mice

GFR was also measured in a subgroup of mice using the MediBeacon Transdermal Mini GFR Monitor System (Mannheim, Germany) as previously described (58). On *day 28*, mice were briefly anesthetized with isoflurane (1.5–2% induction, 1–1.5% maintenance) before placement of transdermal GFR monitors on the flank region using a double-sided adhesive patch (MediBeacon device) (59). The transdermal device was secured using medical adhesive tape. FITC-sinistrin (5 mg/100 g body wt) was administered intravenously via retro-orbital injection. GFR was measured over a 90- to 120-min period in conscious mice with access to food and water ad libitum. The device was then removed, and data were analyzed using elimination kinetics of FITC-sinistrin clearance in MediBeacon MB Lab2 software.

### Isolation and Purification of Podocytes

Podocytes were isolated using previously published methods (60). Briefly, kidneys isolated from KO, HT, and WT male and female mice were minced into small pieces with two scalpels and digested with 2 mg/mL of collagenase (Wako, Osaka, Japan) in complete RPMI-1640 medium containing penicillin, streptomycin, and 5% fetal calf serum (FCS) at 37°C for 40 min with mild rotation. The specimens were then passed through a 100-μm cell strainer (BD Biosciences, San Jose, CA) with a flattened pestle and treated with ammonium-chloride-potassium (ACK) lysis buffer to remove red blood cells. After washing with complete medium, further digestion was performed with 0.5 mg/mL of collagenase and dispase II (Sankojunyaku, Tokyo, Japan) and 0.075% trypsin in complement medium at 37°C for 20 min. Single cells were obtained by passing the cell suspension through a 25-μm filter to remove debris and incompletely dissociated tissue. For the purification of podocytes, single cells (1 × 10^7^) were incubated with 2.5 μg of biotin anti-CD31 monoclonal antibody (BioLegend, San Diego, CA) and Streptavidin MicroBeads (Miltenyi Biotec, Bergisch Gladbach, Germany), and CD31-positive endothelial cells were excluded using the octo-MACS system with MS columns (Miltenyi). The CD31-negative fractions passed through the magnetic columns were subjected to the second MACS separation system using an anti-nephrin antibody (Bioss Antibodies, Woburn, MA). The cells were labeled with 10 μg of biotin-conjugated antibody recognizing the extracellular region of nephrin, followed by another incubation with Streptavidin MicroBeads and separation using the octo-MACS system. The positive and negative fractions of the second MACS separation system were collected as nephrin-positive (podocyte cells) and nephrin-negative (non-podocyte cells) fractions, respectively.

### Real-Time RT-PCR Analysis

Total RNA was extracted from CD31-positive, podocyte, and non-podocyte cells using the RNeasy Plus Mini Kit (Qiagen, Valencia, CA). First-strand cDNA was synthesized from 1 µg of total RNA in a final volume of 20 µL using the RT^2^ First Strand kit. Primers for *Npr1* and glyceraldehyde-3-phosphate dehydrogenase (GAPDH) were purchased from SA Biosciences (Frederick, MD). *Npr1* mRNA was amplified using forward (5′-atctatttcagtgatatcgtgggc-3′) and reverse (5′-catcgaactcttccagcacag-3′) primers. GAPDH was used as an internal control with forward (5′-tccctcaagattgtcagcaa-3′) and reverse (5′-agatccacaaacggatacatt-3′) primers. Quantitative real-time RT-PCR was performed using the Mx3000P real-time PCR system, and data were analyzed with MxPro software (Stratagene, La Jolla, CA) as previously described (61). The reaction mixture without template cDNA was used as a negative control. *Npr1* expression values were normalized to GAPDH. The comparative C_t_ value determined the relative expression of the *Npr1* mRNA.

### Western Blot Analysis

Podocyte cells were washed with PBS and lysed in a buffer containing 25 mM HEPES (pH 7.5), 0.05% 2-mercaptoethanol, 1% Triton X-100, 1 mM sodium vanadate, 10 mm sodium fluoride, 0.2 mM phenylmethylsulfonyl fluoride, and 10 mg/mL each of aprotinin and leupeptin. Cell extract was passed through a 1-mL syringe with a 21-gauge needle and centrifuged at 14,000 rpm for 10 min, as previously reported (62). The clear cell lysate was collected and stored at −80°C until used. The protein concentrations of the lysate were estimated using a Bradford protein detection kit (Bio-Rad, Hercules, CA). Cell lysate (5 µg) was mixed with sample loading buffer and resolved by 10% SDS-PAGE. Proteins were electrotransferred onto a polyvinyl difluoride (PVDF) membrane as previously described (63, 64). The membrane was blocked with 1× Tris-buffered saline-Tween 20 (TBST) containing 5% fat-free milk for 2 h at room temperature and incubated overnight at 4°C in TBST containing 5% fat-free milk with NPRA (135 kDa) primary antibody (1:1,000 dilution). The membrane was then treated with a corresponding secondary anti-chicken horseradish peroxidase (HRP)-conjugated antibody (1:5,000 dilutions), washed three times with TBST, and developed using the SuperSignal West Femto Chemiluminescent Substrate Western Blot Detection Reagent Kit. Protein bands were visualized by the enhanced chemiluminescence (ECL) plus detection system, and luminescent signal was detected using the Alpha Innotech Imaging System (San Loreno, CA). Antibodies of NPRA were custom prepared in our laboratory (with paid services from Genway Biotech) as previously described (65, 66). HRP-conjugated antibody (Cat. No. sc-2428, 1:5,000) was purchased from Santa Cruz Biotechnology.

### Immunofluorescence Staining of Podocin and Synaptopodin

Immunofluorescence analysis was performed as previously reported (67). Paraffin-embedded kidney tissue sections (5 μm) were deparaffinized in xylene (3 × 5 min). Slides were washed with PBS for 5 min and then serially rehydrated with 100%, 90%, and 70% ethanol for 5 min each at room temperature. Antigen retrieval was carried out by microwave oven heating at 95°C for 10 min with 10 mM sodium citrate buffer (pH 6.0). Slides were allowed to cool for 30 min and then washed (three 5-min washes) with distilled water, and the sections were blocked in permeabilization solution (5% BSA in 0.4% Triton X-100 in PBS) for 30 min at room temperature. The permeabilization solution was gently tipped off, and slides were incubated overnight at 4°C with FITC-labeled goat anti-podocin and goat anti-synaptopodin antibodies (1:200 dilution) in PBST containing 5% BSA. Finally, the slides were washed in PBST two times for 10 min each and mounted using a mounting medium containing DAPI (Vector Laboratories). Fluorescence images were obtained in a blinded and unbiased manner under a fluorescence microscope (Olympus BX53) with integrated Magnafire Digital Firewire Camera Software. The antibody-positive area relative to the total glomeruli was calculated using Image-Pro Plus Image Analysis Software (Media Silver Spring, MD). A violet filter was used for DAPI and green filter for FITC. The primary antibodies used were podocin polyclonal antibody FITC conjugated (Cat. No. bs-6597R-FITC, 1:200) obtained from Bioss Antibodies and synaptopodin polyclonal antibody (D9) FITC obtained (Cat. No. sc-515842-FITC, 1:200) from Santa Cruz Biotechnology.

### Statistical Analysis

The statistical analysis was performed using GraphPad Prism Software version 10 (GraphPad Software, San Diego, CA). Group (HT, KO, and WT mice) comparisons and comparisons between multiple groups with two or more experimental factors (for salt diet and sex) were performed using one-way and two-way ANOVA, followed by post hoc analysis with Tukey’s multiple pairwise comparison test. Results are shown as means ± SE, and statistical significance was set at *P* < 0.05.

## RESULTS

### Generation and Validation of Conditional Podocyte-Specific *Npr1* Knockout Mice

**Figure 2.**
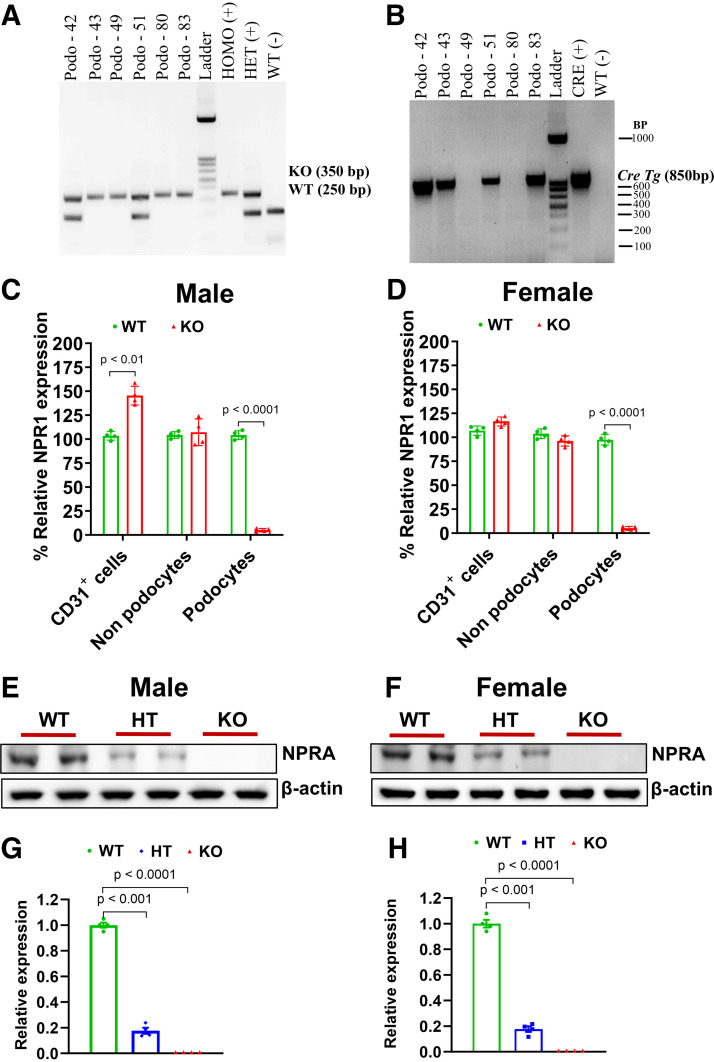
Characterization of the deletion of *Npr1* in podocytes in mice. *A* and *B*: genotypic analysis of podocyte (PD)-specific heterozygous (HT), knockout (KO), wild-type (WT), and *Cre* transgene. DNA was prepared from tail biopsies, and PCR was performed using tail DNA. *A* and *B* show the screening of PD-specific KO and PD-specific *Cre* transgene. The WT band was separated as 250 bp and the KO band as 350 bp. The PD-specific *Cre* transgene was separated as 850 bp. *C* and *D*: absence of expression of *Npr1* mRNA in podocytes of PD-*Npr1* KO mice. Kidney cell suspension of male and female mice was passed through a 70-µm cell strainer (BD Bioscience). Podocytes were purified using magnetic separation columns (Miltenyi). Three fractions of cells (CD31^+^ cells, non-podocytes, and podocytes) were isolated using three-color flow cytometry with fluorescent-labeled antibodies. qPCR measured mRNA expression in each cell type. *E* and *F*: Western blot analysis of NPRA protein levels in podocytes from control and podocyte-specific *Npr1* disrupted male and female mice. *G* and *H*: relative expression of NPRA as analyzed by densitometric analysis. Values are expressed as means ± SE. *n* = 4 preparations using 8 mice/group. Statistically significant *P* values are shown on the graphs. HT, heterozygous; KO, knockout; WT, wild-type.

Genetic identification of *Npr1* mutant and WT alleles with podocyte-specific HT and KO mice was determined by qPCR analysis (Fig. 2, *A* and *B*). The WT band was defined as 250 bp and the KO band as 350 bp. The podocyte-specific *Cre* transgene was defined as 850 bp. To identify *Npr1* mRNA in the PD-*Npr1* KO mice, podocytes were isolated and purified using an Octo-MACS system with MS columns (Miltenyi). The qRT-PCR analysis showed that *Npr1* mRNA was totally absent in podocytes isolated from PD*-Npr1* KO male and female mice; however, *Npr1* mRNA expression was detected in CD31-positive cells, non-podocytes, and podocyte cells in both male and female WT mice (Fig. 2, *C* and *D*). The *Npr1* mRNA level was significantly (*P* < 0.01) higher in CD31-positive cells from PD*-Npr1* KO male mice than in female mice, exhibiting a sex-specific difference in *Npr1* expression in these cells (Fig. 2, *C* and *D*). Western blot analysis showed that NPRA protein was completely absent in the isolated podocytes from PD-*Npr1* KO male and female mice compared with podocytes isolated from WT mice (Fig. 2, *E* and *F*). Densitometry analysis showed that the NPRA protein was not detected in the podocytes isolated from either male or female PD-*Npr1* KO mice but was present in podocytes isolated from WT mice (Fig. 2, *G* and *H*). In podocytes isolated from PD-*Npr1* HT mice, the NPRA protein level was decreased by 61% in males and by 65% in females.

### Effect of Normal-, Low-, and High-Salt Diets on Body Weight, Heart Weight, Food Intake, and Urine Volume in PD-*Npr1* Male and Female Mice

With all three salt diets, heart weight was significantly (*P* < 0.05; *P* < 0.01) increased in PD-*Npr1* HT (male NS: 4.6 ± 0.6, LS: 4.6 ± 0.3, and HS: 5.1 ± 0.4 mg/g body wt; female NS: 4.5 ± 0.4, LS: 4.6 ± 0.4, and HS: 4.8 ± 0.4 mg/g body wt) and KO (male NS: 5.3 ± 0.4, LS: 4.9 ± 0.6, and HS: 5.3 ± 0.2 mg/g body wt; female NS: 4.8 ± 0.2, LS: 4.8 ± 0.7, and HS: 5.0 ± 0.1 mg/g body wt) mice compared with WT (male NS: 4.4 ± 0.3, LS: 4.3 ± 0.5, and HS: 4.5 ± 0.4 mg/g body wt; female NS: 4.3 ± 0.4, LS: 4.2 ± 0.4, and HS: 4.4 ± 0.5 mg/g body wt) mice (Table 1). On the HS diet, the heart weight of both male and female KO mice was increased; however, the magnitude of increase in heart weight in males was significantly (*P* < 0.01) higher compared with females fed the HS diet. No significant differences were observed in the body weight of PD-*Npr1* HT (male NS: 29.3 ± 0.3, LS: 28.7 ± 0.8, and HS: 29.4 ± 0.6 g body wt; female NS: 24.2 ± 0.5, LS: 23.9 ± 0.7, and HS: 23.7 ± 0.7 g body wt) or KO (male NS: 28.8 ± 0.5, LS: 29.4 ± 0.4, and HS: 30.1 ± 0.3 g body wt; female NS: 25.6 ± 0.2, LS: 24.6 ± 0.4, and HS: 24.8 ± 0.3 g body wt) mice compared with WT (male NS: 28.4 ± 0.7, LS: 29.4 ± 0.6, and HS: 28.6 ± 0.7 g body wt; female NS: 23.6 ± 0.6, LS: 23.5 ± 0.5, and HS: 23.8 ± 0.4 g body wt) mice on NS, LS, or HS diets (Table 1). On NS and LS diets, food intake was similar in male and female mice; however, HS diet-fed mice showed significantly (*P* < 0.01; *P* < 0.001) reduced food intake compared with NS and LS diet-fed mice (Table 1). Urine volume was markedly (*P* < 0.05; *P* < 0.01) lower in the PD-*Npr1* HT and KO male and female mice compared with WT mice on NS, LS, and HS diets (Table 1).

**Table 1.** Body weight, heart weight, food intake, and urine volume in podocyte-specific Npr1-disrupted male and female mice fed a normal-, low-, and high-salt diets

Parameters	WT	HT	KO
*Normal salt*			
Body weight, g			
Male	28.4 ± 0.7	29.3 ± 0.3	28.8 ± 0.5
Female	23.6 ± 0.6	24.2 ± 0.5	25.6 ± 0.2
Heart weight, mg/g body wt			
Male	4.4 ± 0.3	4.6 ± 0.6*	5.3 ± 0.4**
Female	4.3 ± 0.4	4.5 ± 0.4*	4.8 ± 0.2**
Food intake, g/24 h			
Male	4.62 ± 0.4	4.3 ± 0.6	4.95 ± 0.5
Female	4.02 ± 0.3	4.11 ± 0.4	4.15 ± 0.3
Urine volume, mL/24 h/g body wt			
Male	0.13 ± 0.01	0.10 ± 0.008*	0.08 ± 0.006**
Female	0.12 ± 0.01	0.10 ± 0.007*	0.08 ± 0.005**
*Low salt*			
Body weight, g			
Male	29.4 ± 0.6	28.7 ± 0.8	29.4 ± 0.4
Female	23.5 ± 0.5	23.9 ± 0.7	24.6 ± 0.4
Heart weight, mg/g body wt			
Male	4.3 ± 0.5	4.6 ± 0.3*	4.9 ± 0.6**
Female	4.2 ± 0.4	4.6 ± 0.4*	4.8 ± 0.7**
Food intake, g/24 h			
Male	4.53 ± 0.5	4.6 ± 0.4	4.34 ± 0.3
Female	4.06 ± 0.4	4.22 ± 0.5	4.16 ± 0.5
Urine volume, mL/24 h/g body wt			
Male	0.12 ± 0.009	0.10 ± 0.007*	0.09 ± 0.005**
Female	0.11 ± 0.008	0.09 ± 0.009*	0.08 ± 0.006**
*High salt*			
Body weight, g			
Male	28.6 ± 0.7	29.4 ± 0.6	30.1 ± 0.3
Female	23.8 ± 0.4	23.7 ± 0.7	24.8 ± 0.3
Heart weight, mg/g body wt			
Male	4.5 ± 0.4	5.1 ± 0.4*	5.3 ± 0.2**
Female	4.4 ± 0.5	4.8 ± 0.4*	5.0 ± 0.1**
Food intake, g/24 h			
Male	3.41 ± 0.4	3.7 ± 0.3	3.88 ± 0.5
Female	2.78 ± 0.3	2.9 ± 0.2	2.95 ± 0.4
Urine volume, mL/24 h/g body wt			
Male	0.24 ± 0.013	0.21 ± 0.011*	0.18 ± 0.012**
Female	0.22 ± 0.014	0.20 ± 0.01*	0.17 ± 0.01**

Values are expressed as means ± SE; *n* = 8 animals in each group. WT, wild-type; HT, heterozygous/ haplotype; KO, knockout. Statistically significant *P* values are indicated as *P < 0.05, **P < 0.01, ***P < 0.001.

### Genetic Deletion of *Npr1* From Podocytes Increases Mean Arterial Pressure

**Figure 3.**
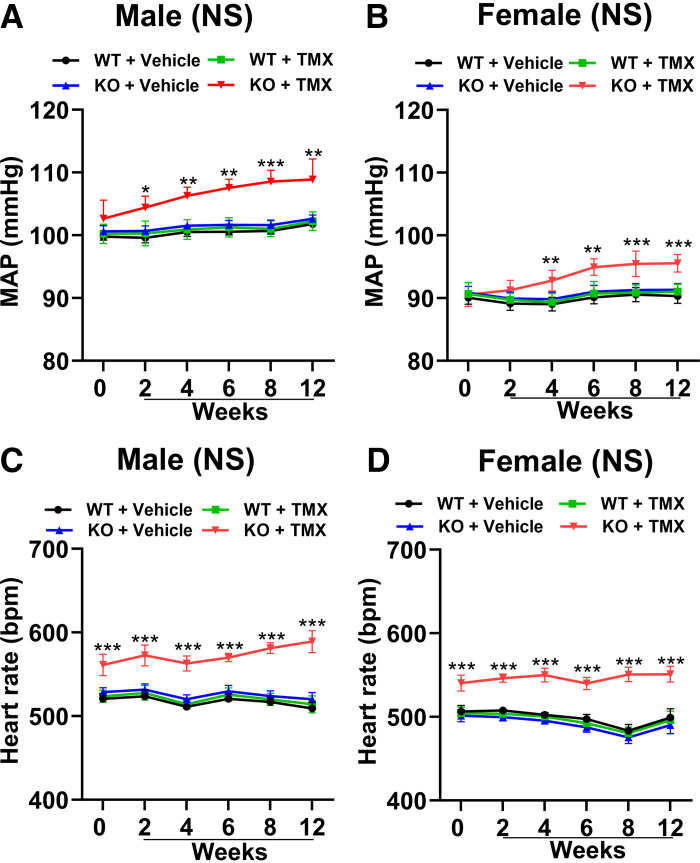
Analysis of mean arterial pressure (MAP) and heart rate (HR) in podocyte (PD)-specific *Npr1*-disrupted male and female mice fed with a normal-salt (NS) diet. BP was measured by radiotelemetry methods. *A* and *B*: MAP in male and female mice fed the NS diet. *C* and *D*: HR in male and female mice fed the NS diet. Values are expressed as means ± SE. *n* = 8 animals in each group. Statistical significance is expressed as *P < 0.05, **P < 0.01, and ***P < 0.001, *Npr1* WT vs. PD-*Npr1* KO mice with the NS diet. bpm, beats/min; KO, knockout; WT, wild-type.

**Figure 4.**
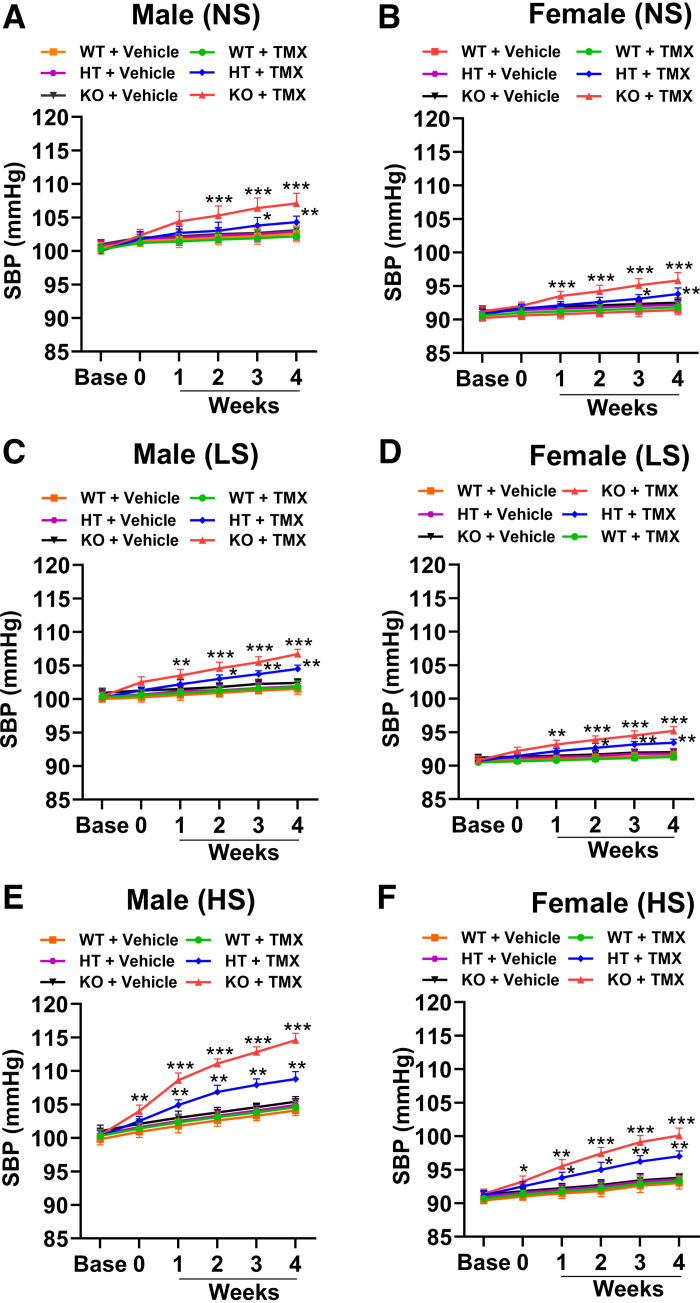
Analysis of systolic blood pressure (SBP) in podocyte (PD)-specific *Npr1*-disrupted male and female mice fed with normal-salt (NS), low-salt (LS), and high-salt (HS) diets. BP was measured using a noninvasive tail-cuff method. *A* and *B*: SBP in male and female PD-*Npr1* WT, HT, and KO mice fed with the NS diet. *C* and *D*: SBP in all three genotypes of PD-*Npr1* mice fed the LS diet. *E* and *F*: SBP in all three genotypes of PD-*Npr1* mice fed the HS diet. Values are expressed as means ± SE. *n* = 8 animals in each group. Statistical significance is expressed as *P < 0.05, **P < 0.01, and ***P < 0.001, PD-*Npr1* WT vs. HT and KO mice with the same salt diet. HT, heterozygous; KO, knockout; WT, wild-type.

On the NS diet, MAP was significantly higher (*P* < 0.05; *P* < 0.01; *P* < 0.001) in both male and female PD-*Npr1* KO mice compared with WT mice at 2, 4, 6, 8, and 12 wk as measured by radiotelemetry (Fig. 3*A*). However, PD-*Npr1* KO male mice developed significantly higher MAP (109 ± 3 mmHg) than female mutant mice (96 ± 1 mmHg) at 12 wk (Fig. 3*B*), although it should be noted that during control (*week 0*) the resting basal level of MAP was higher in male mice. Heart rate (HR) was increased with similar patterns in KO male (589 ± 13 beats/min) and female (551 ± 9 beats/min) mice compared with WT mice (male: 514 ± 10 beats/min; female: 496 ± 10 beats/min), and males had slightly higher levels than females (Fig. 3, *C* and *D*). On NS, LS, and HS diets, SBP levels were significantly (*P* < 0.05; *P* < 0.01; *P* < 0.001) increased in PD-*Npr1* HT and KO male and female mice compared with WT mice at 4 wk as measured by tail cuff (Fig. 4, *A* and *F*). On the HS diet, the magnitude of the increase in SBP at 4 wk was significantly (*P* < 0.01; *P* < 0.001) higher in PD-*Npr1* HT mice (male:109 ± 1 mmHg; female: 97 ± 1 mmHg) and KO mice (male:115 ± 1 mmHg; female: 100 ± 1 mmHg) compared with WT mice (male: 105 ± 1 mmHg; female: 93 ± 1 mmHg) (Fig. 4, *E* and *F*). Although basal SBP (Base) was higher in male mice, the magnitude of the SBP elevating effect was clearly higher in PD-*Npr1*-KO male mice compared with female mutant mice, which may be consequent to sex-specific differences in BP regulation. To exclude the effect of tamoxifen on BP, we measured BP in tamoxifen- and vehicle-treated mice and found it was very similar to levels observed in WT mice treated with vehicle and tamoxifen, indicating that tamoxifen treatment had no influence on BP levels.

### Podocyte-Specific Deletion of *Npr1* Decreased Total Plasma Protein and Increased Urinary Protein Levels in Gene-Disrupted Mice

**Figure 5.**
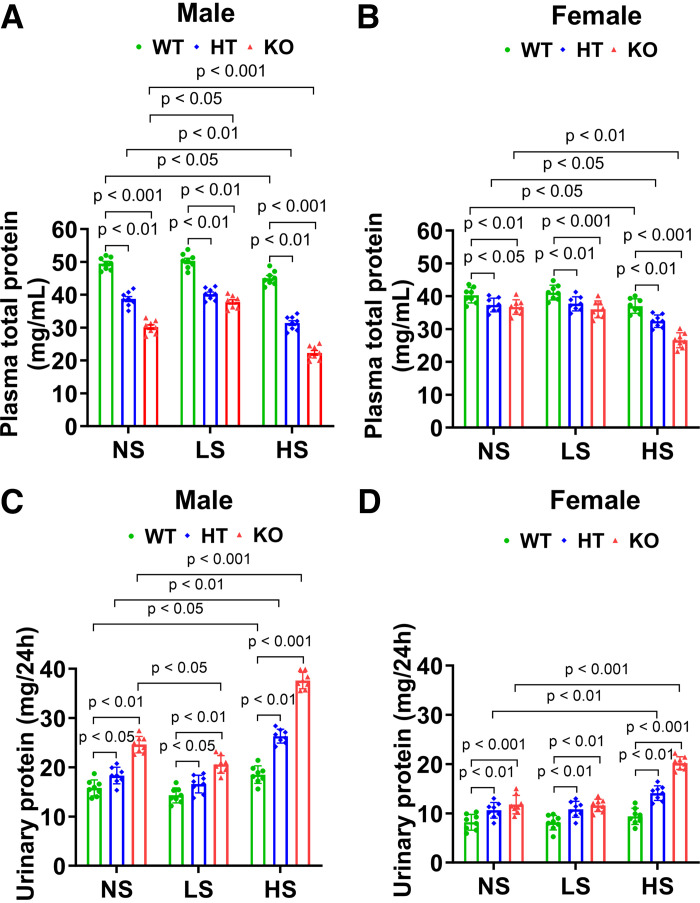
Analysis of plasma total protein and urinary protein in podocyte (PD)-specific *Npr1-*disrupted male and female mice fed with normal-salt (NS), low-salt (LS), and high-salt (HS) diet. *A* and *B*: plasma protein levels in PD-*Npr1* WT, HT, and KO mice fed NS, LS, and HS diet. *C* and *D*: urinary protein levels in all three genotypes of PD-*Npr1* mice fed NS, LS, and HS diets. Values are expressed as means ± SE. *n* = 8 animals in each group. Statistically significant *P* values are shown on the graphs. HT, heterozygous; KO, knockout; WT, wild-type.

After 4 wk of NS, LS, or HS diet, male and female PD-*Npr1* HT (male NS: 38.7 ± 2.2, LS: 40.2 ± 1.8, and HS: 31.4 ± 2.1 mg/mL; female NS: 37.3 ± 2, LS: 37.7 ± 2.1, and HS: 32.4 ± 1.9 mg/mL) and KO (male NS: 30.2 ± 1.9, LS: 37.8 ± 1.9, and HS: 22.3 ± 2 mg/mL; female NS: 36.7 ± 2.2, LS: 36 ± 2.6, and HS: 26.6 ± 2.2 mg/mL) mice showed significantly (*P* < 0.01; *P* < 0.001) decreased plasma total protein levels compared with *Npr1* WT mice (male NS: 49.5 ± 1.9, LS: 50.3 ± 2.2, and HS: 45.1 ± 2.1 mg/mL; female NS: 40.3 ± 2.3, LS: 41.1 ± 2.2, and HS: 37 ± 2.2 mg/mL) (Fig. 5, *A* and *B*). Urinary protein excretion levels were significantly increased (*P* < 0.05; *P* < 0.01; *P* < 0.001) in PD-*Npr1* HT (male NS: 18.3 ± 1.7, LS: 16.6 ± 1.8, and HS: 26.3 ± 1.4 mg/24 h; female NS: 10.6 ± 1.6, LS: 10.8 ± 1.6, and HS: 14.1 ± 1.5 mg/24 h) and KO (male NS: 24.6 ± 1.7, LS: 20.6 ± 1.8, and HS: 37.6 ± 1.7 mg/24 h; female NS: 11.8 ± 1.9, LS: 11.6 ± 1.2, and HS: 20.2 ± 1.3 mg/24 h) mice fed NS, LS, and HS diets compared with WT mice (male NS: 15.8 ± 1.6, LS: 14.3 ± 1.5, and HS: 18.5 ± 1.8 mg/24 h; female NS: 8.2 ± 1.6, LS: 8.1 ± 1.5, and HS: 9.4 ± 1.6 mg/24 h) (Fig. 5, *C* and *D*). These changes were significantly (*P* < 0.01; *P* < 0.001) higher in male and female mice fed a HS diet than those on NS and LS diets. The magnitude of the decrease in plasma total protein and the increase in urinary protein excretion were significantly (*P* < 0.01; *P* < 0.001) greater in male PD-*Npr1* KO mutant mice than in female mutant mice.

### Ablation of Podocyte-Specific *Npr1* Increased Plasma Creatinine and the Albumin/Creatinine Ratio in Gene-Disrupted Mice

**Figure 6.**
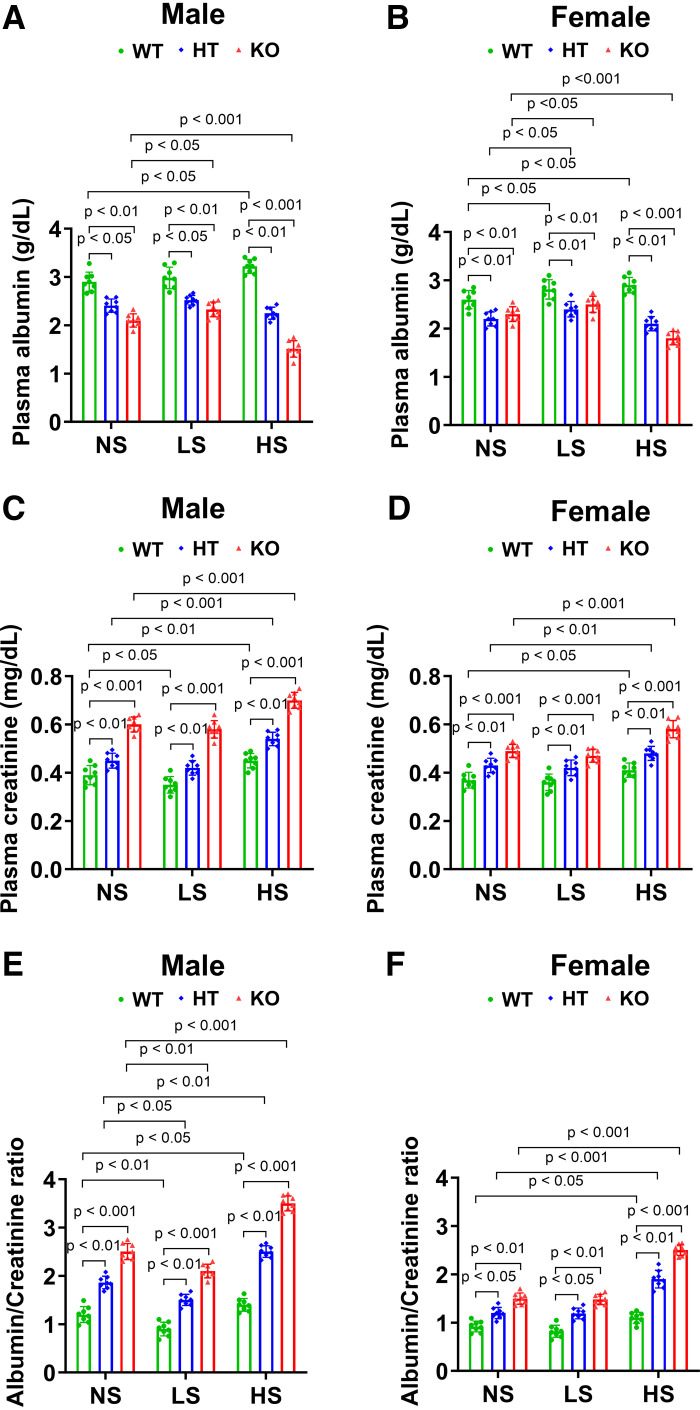
Analysis of plasma albumin, plasma creatinine, and albumin/creatinine ratio in podocyte (PD)-specific *Npr1*-disrupted male and female mice fed with normal-salt (NS), low-salt (LS), and high-salt (HS) diets. *A* and *B*: plasma albumin levels in male and female PD-*Npr1* WT, HT, and KO mice fed NS, LS, and HS diets. *C* and *D*: plasma creatinine levels in all three genotypes kept on NS, LS, and HS diets. *E* and *F*: albumin/creatinine ratio in all three genotypes fed NS, LS, and HS diets. Values are expressed as means ± SE. *n* = 8 animals in each group. Statistically significant *P* values are shown on the graphs. HT, heterozygous; KO, knockout; WT, wild-type.

On NS, LS, and HS diets, plasma albumin levels were significantly (*P* < 0.05; *P* < 0.01; *P* < 0.001) decreased in PD-*Npr1* HT (male NS: 2.4 ± 0.13, LS: 2.5 ± 0.1, and HS: 2.3 ± 0.12 g/dL; female NS: 2.2 ± 0.14, LS: 2.4 ± 0.15, and HS: 2.1 ± 0.14 g/dL) and KO (male NS: 2.1 ± 0.14, LS: 2.3 ± 0.15, and HS: 1.5 ± 0.17 g/dL; female NS: 2.3 ± 0.15, LS: 2.5 ± 0.17, and HS: 1.8 ± 0.13 g/dL) male and female mice compared with WT mice (male NS: 2.9 ± 0.2, LS: 3 ± 0.22, and HS: 3.2 ± 0.14 g/dL; female NS: 2.6 ± 0.19, LS: 2.8 ± 0.2, and HS: 2.9 ± 0.2 g/dL) (Fig. 6, *A* and *B*). Furthermore, plasma creatinine levels were significantly (*P* < 0.01; *P* < 0.001) increased in PD-*Npr1* HT (male NS: 0.45 ± 0.03, LS: 0.42 ± 0.03, and HS: 0.54 ± 0.02 mg/dL; female NS: 0.43 ± 0.03, LS: 0.42 ± 0.03, and HS: 0.48 ± 0.02 mg/dL) and KO (male NS: 0.6 ± 0.03, LS: 0.58 ± 0.03, and HS: 0.7 ± 0.03 mg/dL; female NS: 0.49 ± 0.02, LS: 0.47 ± 0.02, and HS: 0.58 ± 0.03 mg/dL) male and female mice compared with WT mice (male NS: 0.39 ± 0.04, LS: 0.35 ± 0.03, and HS: 0.45 ± 0.03 mg/dL; female NS: 0.37 ± 0.03, LS: 0.36 ± 0.03, and HS: 0.41 ± 0.02 mg/dL) (Fig. 6, *C* and *D*). Decreases in plasma albumin and increases in plasma creatinine were significantly (*P* < 0.001) higher in male and female mice fed HS diets than those on NS and LS diets (Fig. 6, *A* and *D*). However, the magnitude of these changes in males fed a HS diet was significantly higher (*P* < 0.01) compared with that of females fed a HS diet. The albumin/creatinine ratio was also greatly (*P* < 0.05; *P* < 0.01; *P* < 0.001) increased in male PD-*Npr1* HT (male NS: 1.86 ± 0.12, LS: 1.5 ± 0.11, and HS: 2.5 ± 0.12; female NS: 1.19 ± 0.11, LS: 1.19 ± 0.11, and HS: 1.9 ± 0.18) and KO (male NS: 2.5 ± 0.16, LS: 2.1 ± 0.14, and HS: 3.5 ± 0.15; female NS: 1.49 ± 0.11, LS: 1.48 ± 0.10, and HS: 2.5 ± 0.11) mice than in female mutant mice compared with WT mice (male NS: 1.2 ± 0.16, LS: 0.9 ± 0.14, and HS: 1.4 ± 0.13; female NS: 0.92 ± 0.11, LS: 0.82 ± 0.12, and HS: 1.1 ± 0.11) fed with respective NS, LS, or HS diets (Fig. 6, *E* and *F*), and the albumin/creatinine ratio was significantly lower in females than in males.

### Deletion of Podocyte-Specific *Npr1* Decreased Creatinine Clearance and Glomerular Filtration Rate and Increased Plasma Sodium in Mutant Mice

**Figure 7.**
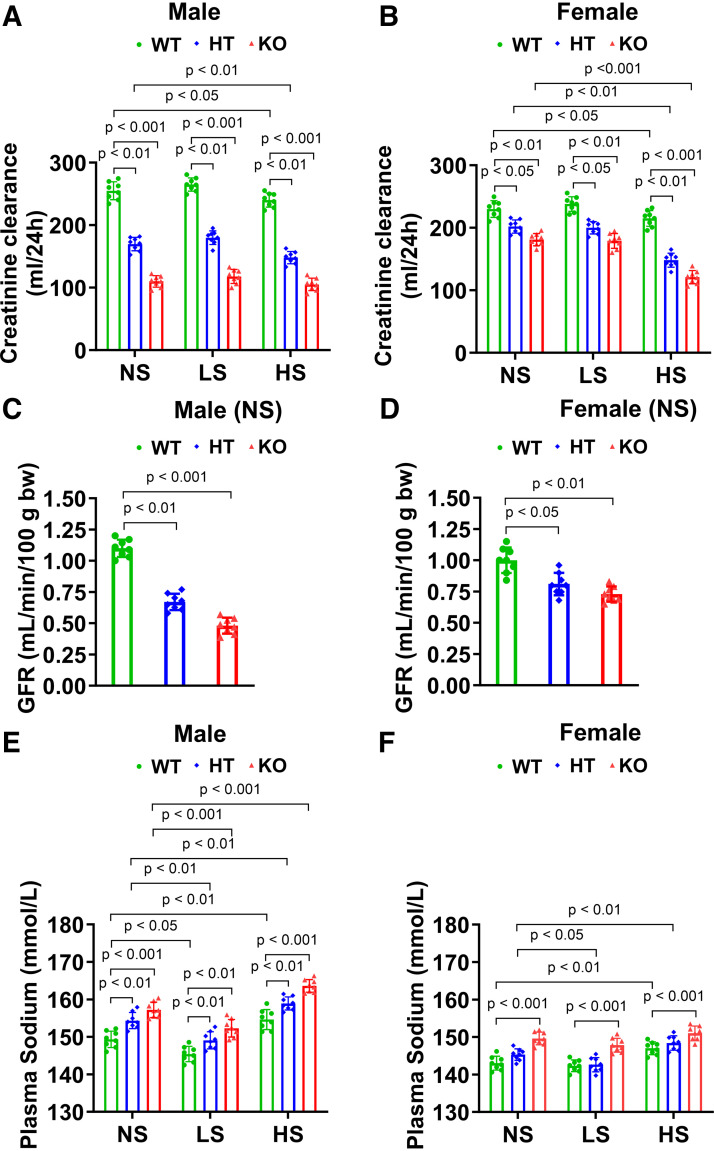
Analysis of creatinine clearance, glomerular filtration rate (GFR), and plasma sodium in podocyte (PD)-specific *Npr1*-disrupted male and female mice fed with normal-salt (NS), low-salt (LS), and high-salt (HS) diets. *A* and *B*: creatinine clearance in PD-specific *Npr1* gene-disrupted mice fed NS, LS, and HS diets. *C* and *D*: analysis of GFR in PD-specific *Npr1*-disrupted male and female mice fed with the NS diet. *E* and *F*: analysis of plasma sodium in PD-specific *Npr1*-disrupted male and female mice fed with NS, LS, and HS diets. Values are expressed as means ± SE. *n* = 8 animals in each group. Statistically significant *P* values are shown on the graphs. HT, heterozygous; KO, knockout; WT, wild-type.

On NS, LS, and HS diets, CrCl levels were greatly (*P* < 0.01; *P* < 0.001) decreased in PD-*Npr1* HT (male NS: 169.8 ± 10.7, LS: 180 ± 10.9, and HS: 148 ± 9.7 mL/24 h; female NS: 202 ± 10, LS: 200 ± 9, and HS: 148 ± 11 mL/24 h) and KO (male NS: 110 ± 9, LS: 118 ± 11, and HS: 105 ± 10 mL/24 h; female NS: 181 ± 9, LS: 179 ± 11, and HS: 121 ± 10 mL/24 h) male and female mutant mice compared with WT mice (male NS: 255 ± 14, LS: 264 ± 10, and HS: 240 ± 11 mL/24 h; female NS: 230 ± 13, LS: 238 ± 11, and HS: 215 ± 12 mL/24 h) (Fig. 7, *A* and *B*). The magnitude of the CrCl reduction was greater in males than in females. GFR levels were significantly (*P* < 0.05; *P* < 0.01; *P* < 0.001) reduced on a NS diet in PD-*Npr1* HT (male: 0.67 ± 0.06 mL/min/100 g body wt; female: 0.81 ± 0.09 mL/min/100 g body wt) and KO (male: 0.48 ± 0.06 mL/min/100 g body wt; female: 0.73 ± 0.06 mL/min/100 g body wt) male and female mice compared with WT mice (male: 1.1 ± 0.07 mL/min/100 g body wt; female: 1 ± 0.1 mL/min/100 g body wt) (Fig. 7, *C* and *D*), but the magnitude of GFR reduction levels was greater (*P* < 0.01) in male mice than in female mice. On NS, LS, and HS diets, PD-*Npr1* HT (NS: 154.3 ± 2.1, LS: 149 ± 2.2, and HS: 159 ± 1.7 mmol/L) and KO (NS: 157.2 ± 2, LS: 152.3 ± 2.3, and HS: 164 ± 1.7 mmol/L) male mice showed significantly (*P* < 0.01; *P* < 0.001) increased plasma sodium levels compared with WT mice (NS: 149.3 ± 2.1, LS: 145.4 ± 2, and HS: 154.6 ± 2.6 mmol/L) (Fig. 7*E*). Plasma sodium levels were significantly (*P* < 0.001) increased in PD-*Npr1* KO female mice (NS: 149.6 ± 1.6, LS: 147.8 ± 1.8, and HS: 151 ± 1.9 mmol/L), and no significant differences were noted in HT female mice (NS: 145.3 ± 1.5, LS: 142.6 ± 1.8, and HS: 148.4 ± 1.9 mmol/L) compared with WT mice (NS: 143 ± 1.8, LS: 142.3 ± 1.5, and HS: 147 ± 1.7 mmol/L) fed NS, LS, and HS diets (Fig. 7*F*). The magnitude of the increase in plasma sodium concentrations was greater for HS diet-fed mice and lower for LS diet-fed mice compared with NS diet-fed mice.

### Podocyte-Specific Deficiency of *Npr1* Lowered Urinary Sodium Excretion Levels in Mutant Mice

**Figure 8.**
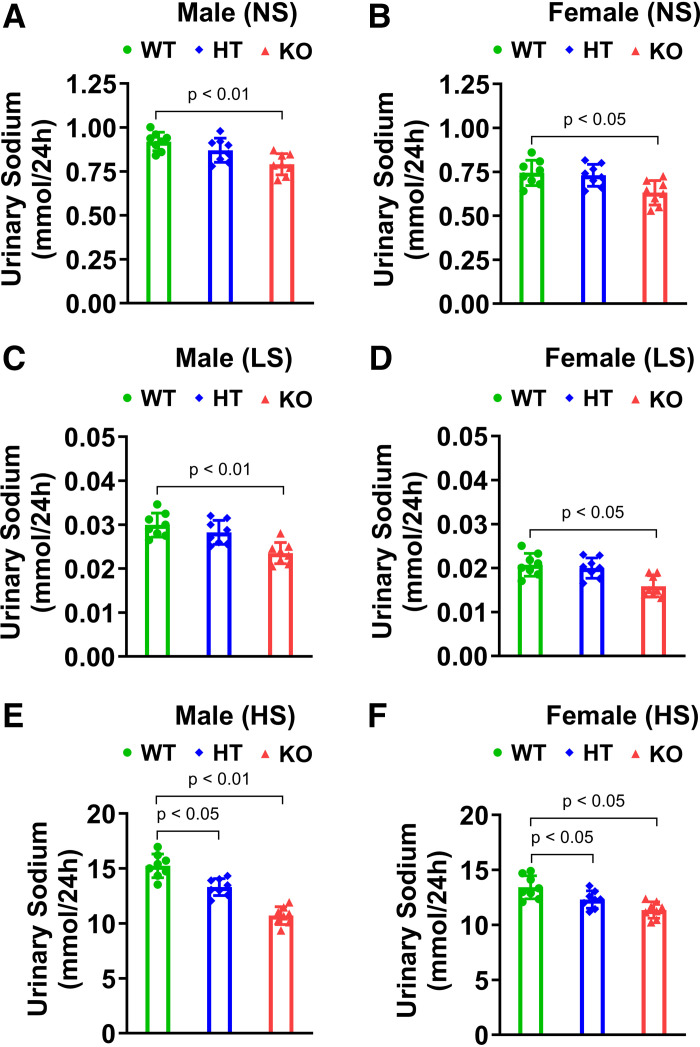
Analysis of urinary sodium in podocyte (PD)-specific *Npr1*-disrupted male and female mice fed with normal-salt (NS), low-salt (LS), and high-salt (HS) diets. *A*–*F*: analysis of urinary sodium in PD-specific gene-disrupted male and female mice fed with NS, LS, and HS diets. Values are expressed as means ± SE. *n* = 8 animals in each group. Statistically significant *P* values are shown on the graphs. HT, heterozygous; KO, knockout; WT, wild-type.

The 24-h urinary sodium excretion levels were markedly decreased in male and female PD-*Npr1* KO mice (male NS: 0.79 ± 0.06 and LS: 0.024 ± 0.002; mmol/24 h female NS: 0.63 ± 0.07 and LS: 0.016 ± 0.002 mmol/24 h), but there were no significant differences in HT male or female mice (male NS: 0.87 ± 0.06 and LS: 0.028 ± 0.003 mmol/24 h; female NS: 0.73 ± 0.06 and LS: 0.020 ± 0.002 mmol/24 h) fed NS and LS diets compared with WT mice (male NS: 0.92 ± 0.05 and LS: 0.03 ± 0.003 mmol/24 h; female NS: 0.744 ± 0.07 and LS: 0.021 ± 0.003 mmol/24 h) (Fig. 8, *A–D*). HS diet-fed PD-*Npr1* HT (male: 13.3 ± 0.77 mmol/24 h; female: 12.3 ± 0.78 mmol/24 h) and KO (male: 10.7 ± 0.80 mmol/24 h; female: 11.35 ± 0.74 mmol/24 h) mice showed noticeable differences (*P* < 0.05; *P* < 0.01), with decreased urinary sodium excretion levels compared with WT mice (male: 15.23 ± 1.08 mmol/24 h; female: 13.42 ± 1.04 mmol/24 h) (Fig. 8, *E* and *F*).

### Ablation of *Npr1* Decreased the Immunofluorescence Intensity of Podocin and Synaptopodin in Podocytes of Mutant Mice

**Figure 9.**
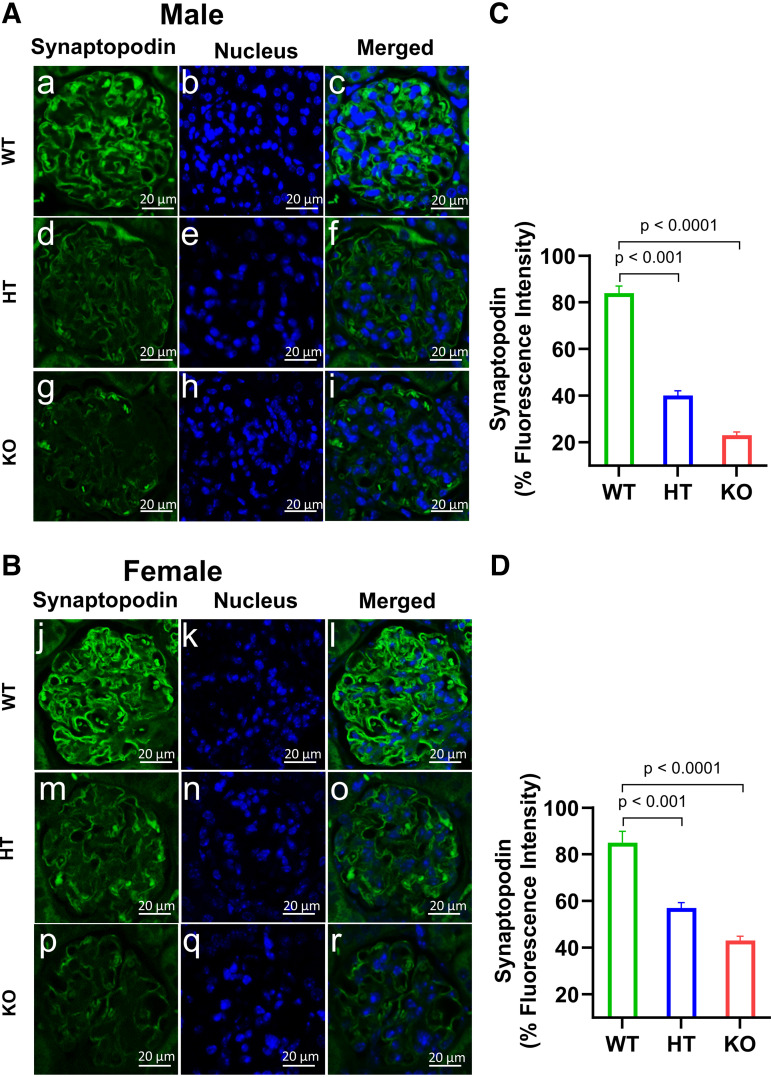
Immunofluorescence analysis of synaptopodin protein in kidney tissues of podocyte-specific *Npr1* gene-disrupted male and female mice fed with the normal-salt diet. *A* and *B*: analysis of synaptopodin immunoreactivity in kidney tissues. *C* and *D*: densitometric quantitative analysis of synaptopodin protein expression levels in kidney tissues of male and female. Values are expressed as means ± SE. *n* = 8 animals in each group. Statistically significant *P* values are shown on the graphs. HT, heterozygous; KO, knockout; WT, wild-type.

**Figure 10.**
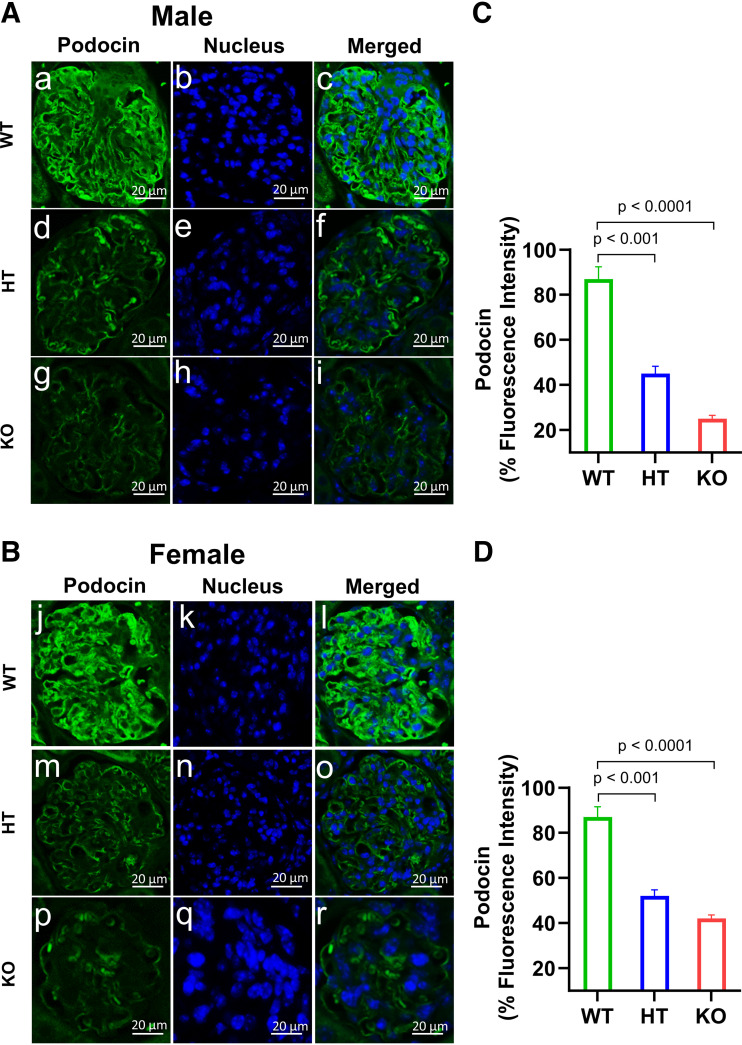
Immunofluorescence analysis of podocin protein in kidney tissues of podocyte-specific *Npr1*-disrupted male and female mice fed with the normal-salt diet. *A* and *B*: analysis of podocin immunoreactivity in kidney tissues. *C* and *D*: densitometric quantitative analysis of podocin protein expression levels in kidney tissues of male and female mice. Values are expressed as means ± SE. *n* = 8 animals in each group. Statistically significant *P* values are shown on the graphs. HT, heterozygous; KO, knockout; WT, wild-type.

To evaluate the integrity of podocytes in the kidneys of PD-*Npr1* mutant mice, we determined the fate of the podocyte marker proteins podocin and synaptopodin by immunofluorescence analysis (Fig. 9, *A* and *B*, and Fig. 10, *A* and *B*). In the kidneys of PD-*Npr1* WT male and female mice, synaptopodin was strongly detected and was distributed evenly among the glomeruli. In contrast, markedly decreased synaptopodin-positive areas were observed in PD-*Npr1* HT and KO male and female mice compared with WT mice (Fig. 9, *A* and *B*). Quantitative measurement showed that the intensity of synaptopodin was greatly (*P* < 0.001; *P* < 0.0001) reduced in the glomeruli of male and female PD-*Npr1* HT and KO mice compared with WT mice (Fig. 9, *C* and *D*). Thus, the degree of decrease in immunostaining of synaptopodin was higher in PD-*Npr1* HT and KO male mice than in female mice. The intensity of podocin was also reduced in the glomeruli of PD-*Npr1* HT and KO male and female mice compared with WT mice (Fig. 10, *A* and *B*). Immunofluorescence quantitative analysis indicated that the magnitude of immunostaining of podocin was decreased, but these decreases were significantly (*P* < 0.05) greater in animals male than in female animals (Fig. 10, *C* and *D*).

## DISCUSSION

Our results demonstrate that ablation of the *Npr1* gene led to increased BP and increased levels of plasma creatinine, urinary protein, and albumin/creatinine ratio in PD-*Npr1* HT and KO mice; however, total plasma protein, albumin, and CrCl were significantly reduced in mutant mice compared with WT mice. GFR was significantly decreased in both HT and KO mice compared with WT mice, and these changes were significantly greater in males than in females. GFR is the true indicator of the blood volume that is filtered from the kidney’s glomerular capillaries into Bowman’s capsule per unit of time, as determined by the balance of hydrostatic and colloidal osmotic forces across the glomerular membrane along with its permeability and surface area (68). In the glomerulus, ANP and BNP vasodilate the afferent arterioles and contract the efferent arterioles, increasing glomerular capillary pressure and improving GFR and fractional filtration (16). In this study, GFR levels were decreased in PD-*Npr1* HT and KO mice compared with WT mice, suggesting that ANP/NPRA signaling in podocytes contributes to the regulation of GFR, possibly through an alteration in the filtration barrier. PD-*Npr1* KO female mice showed a significantly greater GFR than their male counterparts, indicating that the female sex hormones could have a protective effect. Another study using male PD-GC-A KO mice reported that NPRA in podocytes is renoprotective, but its role seems to be nonessential to renal hemodynamic functions (69). Those previous studies showed that under basal conditions, PD-GC-A KO mice and control WT mice did not show differences in BP, GFR, or natriuresis; however, the administration of mineralocorticoid deoxycorticosterone (DOCA) and high salt intake increased BP in both PD-GC-A KO and WT mice. The combination of DOCA and HS diet led to an increase in mild albuminuria and slightly decreased GFR, but only in PD-GC-A KO mice, compared with controls. A significant residual expression of *Npr1* mRNA was observed in those PD-GC-A *Npr1* KO mice (69). The authors used a conditional targeting vector to delete only the exon 1 region of the *Npr1* construct, which may explain the discrepancies between our studies (69). In the current study, we targeted both exon 1 and exon 2 regions to construct the targeting vector to produce PD-*Npr1* KO mice. Our present findings contradict previous results suggesting that the deletion of *Npr1* in podocytes exhibits a direct impact on BP and GFR in PD-*Npr1* mutant mice in a sex-dependent manner. We observed significant differences in BP, GFR, and urinary sodium levels in PD-*Npr1* HT and KO mice compared with WT mice under basal conditions, and these effects were more prominent in PD-*Npr1* HT and KO mice on a HS diet compared with control groups and more pronounced in males than in females in a sex-dependent manner.

The podocyte marker proteins synaptopodin and podocin were also greatly reduced in PD-*Npr1* HT and KO mice compared with WT mice. This suggests that in podocytes, ANP/NPRA/cGMP signaling is crucial in the maintenance and regulation of BP and GFR and represents a critical biomarker of BP and renal hemodynamic function. To determine the exact role of *Npr1* in podocytes in a sex-specific manner, we used both male and female PD-*Npr1* KO mice in this study. High salt intake is associated with adverse health outcomes related to increased BP (70, 71). Salt plays an essential role in the progression of chronic kidney disease (CKD), in which decreased GFR characterizes albuminuria as well as glomerular and tubular-interstitial fibrosis in BP-dependent and independent manners (72, 73). In clinical settings, elevated BP with relatively high salt intake is defined as salt-sensitive hypertension (SSHT) (74). In 60% of patients with hypertension, increased salt intake is associated with uncontrollable BP (75). Loss of podocytes, glomerular damage, and proteinuria are among the primary signs of kidney disease in SSHT (76–78). Hypertension-induced renal injury, including nephrosclerosis, exhibits a combination of pathological changes in the glomerulus and tubulointerstitium, and, in most cases, the extent of kidney injury reflects the severity and duration of high BP. Hypertensive nephrosclerosis progresses slowly and is associated with fibrinoid necrotic cell growth and proliferation that may lead to end-stage renal disease (ESRD). In the present study, SBP levels were significantly elevated in PD-*Npr1* HT and KO male and female mice compared with WT mice on NS, LS, and HS diets. SBP increased by 4 mmHg in HT and 10 mmHg in KO male mice and 4 mmHg in HT and 7 mmHg in KO female mice compared with WT mice on a HS diet. We found that PD-*Npr1*-HT and KO male mice developed a higher SBP than female mice on a HS diet. The differences in BP between male and female mice are indicative of sex-specific differences due to the loss of *Npr1* in podocytes. In this study, heart weight was increased in PD-*Npr1* HT and KO male and female mice compared with WT mice on a HS diet. It is possible that elevated sympathetic activity may also contribute toward the underlying cause of increased SBP in podocyte-specific *Npr1* mutant mice. Our previous studies have also shown that global 1-copy haplotype *Npr1^+/−^* mice that received a HS diet exhibited increased SBP compared with WT *Npr1^+/+^* animals (37, 38). HT mice expressing one *Npr1* allele showed that SBP was significantly increased in animals fed on NS, LS, and HS diets, indicating that reduced *Npr1* expression in podocytes leads to elevated SBP, and that in podocytes, *Npr1* is crucial in regulating SBP and renal function on a HS diet. Likewise, the SBP elevating effect of a HS diet was greater in male PD-*Npr1* KO mice compared with female mice, suggesting that female mice possess protective mechanisms against high salt-induced increases in BP. MAP was significantly increased in PD-*Npr1* KO male and female mice compared with *Npr1* WT mice on a NS diet, suggesting that ablation of *Npr1* in podocytes impairs BP regulation.

Evidence from clinical and experimental studies indicates that albuminuria and proteinuria are not simply markers of CKD progression but active players in the evolution of the disease process (79). Podocyte injury is considered the most important early event in the pathogenesis of proteinuria and progressive glomerulosclerosis in patients with proteinuric kidney diseases (80). Mechanistically, it has been proposed that proteins that escape into the glomerular filtrate have a toxic effect on tubular cells, and these damaged tubular cells may lead to the development and progression of inflammation and interstitial fibrosis (64, 81). In podocytes, cGMP regulates the filtration barrier by modulating the slit membrane and cytoskeletal protein reorganization (82, 83). Impaired function or depletion of podocytes contributes to many forms of proteinuric CKD (84). A recent study reported that KO mice lacking the podocyte-specific tight junction integral membrane protein claudin-5 exhibit podocyte injury and proteinuria with diabetic nephropathy (85). Numerous studies have shown that proteinuria is significantly associated with certain kidney functional metrics, including serum creatinine level, GFR, and progression to ESKD (86). Proteinuria may contribute to disease progression through a number of mechanisms, including direct renal cell toxicity, glomerular overload, specific renal filtered proteins, and induction of damaged protein molecules. In our study, high salt intake was associated with increased urinary loss of proteins and decreased plasma proteins, suggesting that ablation of *Npr1* signaling may damage the ultrafiltration barrier and could lead to glomerular proteinuria. Estrogens are known to provide protective effects against renal disorders in females (87). We observed that the magnitude of increased proteinuria in PD-*Npr1* KO males fed a HS diet was significantly higher compared with that of PD-*Npr1* KO females fed a HS diet. We expect that estrogens may provide protection for female mice against proteinuria induced by podocyte-specific *Npr1* gene disruption in female animals; however, the exact roles of sex hormones are unclear and warrant further investigation.

In the present study, the decrease in plasma albumin and increase in the urinary albumin concentrations of HS diet-fed PD-*Npr1* HT and KO mice indicate the onset of albuminuria and renal dysfunction. It is possible that PD-*Npr1* KO mice fed a HS diet are particularly sensitive to its harmful effects, with the selective permeability of the GBM causing higher urinary excretion of albumin with high salt intake. A previous study indicated that podocyte-specific pyruvate kinase M2 KO mice showed aggravated albuminuria and pathological severity of CKD progression (88). Interestingly, albuminuria is known to decrease significantly after treatment with ANP in male and female mice in a sex-dependent manner (89). We suspect that the increased albuminuria observed in PD-*Npr1* HT and KO male and female mice in this study may be due to the disruption of ANP-BNP/NPRA/cGMP signaling cascade in podocytes.

Creatinine is formed in muscle from creatine phosphate by irreversible, nonenzymatic dehydration and loss of phosphate contents. Serum levels and renal CrCl indicate proper renal function, as creatinine is excreted only via the kidneys (90). In the present study, on a HS diet, PD-*Npr1* HT and KO male mice developed higher plasma creatinine levels and reduced CrCl compared with NS and LS diet-fed groups. Female mutant mice also showed relatively higher plasma creatinine levels but to a much lower degree than males. Significant increases in plasma creatinine concentrations and decreased CrCl rates suggest the onset of renal insufficiency with podocyte-specific disruption of *Npr1*. It has been suggested that the pharmacological generation of second messenger cGMP by sildenafil may have a direct effect on renal hemodynamics by preventing glomerular hypertension and hyperfiltration (91). We suspect that these mechanisms might have contributed to the increased plasma creatinine and decreased CrCl observed in PD-*Npr1* HT and KO mice. CrCl levels were higher in PD-*Npr1* KO female mice on all three diets than in male mice. Likewise, the magnitude of the plasma creatinine elevating effect of a HS diet was higher in PD-*Npr1* KO male mice compared with their female counterparts, likely due to the renal protective role of sex hormones in females.

In the current study, the increase in plasma sodium concentration in HS diet-fed PD-*Npr1* KO mice suggests that Na^+^ retention increases extracellular fluid volume and preload to increase BP. Sodium excretion was reduced in the HS diet-fed PD-*Npr1* KO male and female mice. These findings are consistent with our earlier reports that salt loading leads to decreased urinary sodium excretion in global *Npr1* gene-disrupted female mice, suggesting that low ANP/NPRA signaling might lead to sodium retention and elevated BP in mutant mice (37, 38). Excess dietary sodium intake over the renal excretory capacity leads to an osmotically driven expansion of extracellular fluid (ECF) volume. Salt loading increases ECF volume and cardiac preload, which may lead to higher pulse volume; however, if the kidney loses the ability to excrete excessive salt and water, pulse volume might also be increased and may lead to an elevation in BP (92). We have previously demonstrated that global gene *Npr1*-disrupted heterozygous (1-copy) mice fed a HS diet showed elevated SBP and aggravated levels of cardiac ANG II, whereas *Npr1* gene-duplicated mice did not exhibit such elevated effects (37, 38). Our previous studies also demonstrated essential roles for GC-A/NPRA signaling in acute volume handling and protecting the heart from salt loading through the kidney (39–42).

We observed a markedly reduced localization of the SD protein podocin and the foot process cytoskeleton protein synaptopodin in podocytes of PD-*Npr1* HT and KO mice. Nephrotic syndrome studies in humans have indicated that these proteins play essential roles in maintaining the structural and functional integrity of the SD. Nephrotic syndrome conditions include an increased permeability of the GFB with proteinuria accompanied by consequent edema, hypoalbuminemia, and hyperlipidemia (93). It has been suggested that podocin regulates the structural organization and filtration function of the SD by interacting directly with nephrin; however, depletion of podocin contributes to pathological filtration that may lead to severe proteinuria (94, 95). Synaptopodin is an actin-associated protein that is linked to the formation of foot processes, a hallmark of the differentiated podocyte phenotype and its function (96, 97). We found significantly reduced immunofluorescence, expression, and localization of podocin and synaptopodin in kidney sections of PD-*Npr1* HT and KO male and female mutant mice, which is also aligned with increased proteinuria. In male PD-*Npr1* KO mice, the immunofluorescence intensity of both podocin and synaptopodin was markedly lower compared with females. A recent study demonstrated that podocyte-specific tubular sclerosis complex 2 deletion in mice contributes to severe podocyte injury, leading to massive proteinuria, end-stage renal dysfunction, and decreased immunofluorescence intensity of podocin and synaptopodin (98).

In its early stages, podocyte injury is correlated with a decreased expression of podocin, synaptopodin, and nephrin, leading to cytoskeleton disorders, foot process fusion, and proteinuria (99). This study found that CD31-positive cells showed increased expression of *Npr1* in a sex-dependent manner in PD-*Npr1* KO mice. The cell adhesion molecule CD31 is typically used as an endothelial cell marker to detect angiogenesis. It transmits downstream signaling during cell-cell interaction, which plays a pivotal role in maintaining GFB involving both podocyte and endothelial cells (100, 101). During glomerular injury, the CD31-positive^+^ cell type may undergo specific alterations in cellular morphology and function, affecting glomerular filtration. Podocytes are attached to the exteriors of the GBM alongside endothelial cells and serve as the barrier in preventing proteinuria (102). CD31-positive cells may compensate for damaged podocytes in PD-*Npr1* KO mice to help reduce proteinuria. The expression of *Npr1* in CD31-positive cells might provide a mechanism to increase the filtration surface area to compensate for the glomerular damage caused by leaked proteins from the deficiency in podocin and synaptopodin in PD-*Npr1* KO mice. Our results suggest that podocyte-specific *Npr1* disruption may account for the podocyte damage and reduced expression of podocin and synaptopodin, respectively, that disrupt the functional integrity of the SD and formation of foot processes and may lead to proteinuria in PD-*Npr1* mutant animals.

In conclusion, our findings demonstrate that BP, plasma creatinine, plasma sodium, urinary protein, and albumin/creatinine ratio were significantly increased, whereas plasma total protein, albumin, CrCl, and urinary sodium levels were significantly reduced in the HT and KO male and female mice compared with WT mice. Furthermore, these changes were significantly greater in males than in females. GFR was significantly decreased in PD-*Npr1* HT and KO male and female mice compared with WT mice. Podocyte-specific *Npr1* KO and HT mice fed a HS diet showed more severe increased BP and altered biomarkers of renal function compared with NS or LS diets. On a NS diet, podocyte-specific *Npr1-*disrupted mice exhibited decreased immunofluorescence expression of the SD-associated proteins podocin and synaptopodin, and this effect was greater in males than in females. Future studies should investigate specific mechanisms underlying how podocyte-specific *Npr1*-disrupted female mice are protected under basal and HS conditions compared with males. This study suggests a direct and sex-dependent effect of *Npr1* in podocytes on the regulation of BP and GFR, which reveals that podocytes should be considered an important target for the ANP-BNP/NPRA/cGMP signaling cascade to protect against high BP and kidney dysfunction.

## DATA AVAILABILITY

Data will be made available upon reasonable request.

## GRANTS

This work was supported by National Institute of Diabetes and Digestive and Kidney Diseases Grant DK133833.

## DISCLOSURES

No conflicts of interest, financial or otherwise, are declared by the authors.

## AUTHOR CONTRIBUTIONS

C.R. and K.N.P. conceived and designed research; C.R., K.N., S.R., H.X., D.R.K., F.R.D., and K.N.P. performed experiments; C.R., K.N., and K.N.P. analyzed data; C.R. and K.N.P. interpreted results of experiments; C.R. and K.N.P. prepared figures; C.R. and K.N.P. drafted manuscript; C.R. and K.N.P. edited and revised manuscript; C.R., K.N., S.R., H.X., D.R.K., F.R.D., and K.N.P. approved final version of manuscript.
